# Speech-Language Therapy Through Telepractice During COVID-19 and Its Way Forward: A Scoping Review

**DOI:** 10.7759/cureus.44808

**Published:** 2023-09-06

**Authors:** Ishita Guglani, Sanskriti Sanskriti, Shiv H Joshi, Ashish Anjankar

**Affiliations:** 1 Community Medicine, Jawaharlal Nehru Medical College, Datta Meghe Institute of Higher Education and Research, Wardha, IND; 2 Biochemistry, Jawaharlal Nehru Medical College, Datta Meghe Institute of Higher Education and Research, Wardha, IND

**Keywords:** covid-19, telepractice, speech-language disorder, speech-language pathologists, speech and language therapy

## Abstract

The overall burden of voice disorders is vast, and speech-language therapy has been in use for long to prevent, assess, diagnose, and treat different speech and language disorders. Due to the COVID-19 outbreak, these services are not readily accessible because of various precautionary measures that have been laid down by the government to check the blowout of infection; as a solution to this, there has been a rise in telepractice. The purpose of this review article is to study the usefulness of telepractice for speech-language therapy during the COVID-19 pandemic and its way forward. Search was performed in the PubMed database. A total of 102 articles were found, out of which 32 articles were included through a comprehensive inclusion and exclusion criteria. This study analyzes various papers on the use of telepractice during COVID-19 for speech-language therapy. The satisfaction was greater among women as compared to men because women could get the appointment done at home and they could easily manage their household chores. It has been recognized as an "attend anywhere" web-based platform that provides us with the 5 C's, namely, easy-to-access care, increased comfort, increased convenience, reduced cost, and higher confidentiality. Patients look for such sessions in the future, even when the pandemic is over. Telepractice has now been accepted as the new healthcare delivery model with multiple advantages and disadvantages. However, more research needs to be done on the moral and environmental aspects related to its use.

## Introduction and background

The gift of speech is among the most wonderful things that is bestowed upon mankind, and it is by means of this voice that we are able to communicate with other people all over the world [1]. The speaker's voice, which is woven into the fabric of the speech they deliver, is considered to be their unique identifier [1]. There are some people among us who will never have the opportunity to benefit from this gift of speech and language in their lifetime. Voice and speech-language disorders place a significant and widespread burden on populations all over the world. There are approximately 18.5 million people around the world who are known to be suffering from speech and language disorders, as stated by the National Institute on Deafness and Other Communication Disorders [2]. According to the census completed in 2011, India itself comprises approximately five million people of the total burden. People who have been diagnosed with speech-language disorders can participate in a variety of speech therapies in an effort to alleviate some of the sufferings they experience as a result of having these disorders. Different techniques from the field of speech-language therapy are employed in order to diagnose and successfully treat speech disorders [3]. 

The methods that are applied in the process of treating such conditions changes depending on the patient's age. These methods include articulation therapy, which is administered to children, and accent modification, which is administered to adults. However, due to COVID-19, there has been an interruption in the supply of these face-to-face speech therapies. As a result, there has been a prompt adoption of the innovative healthcare service delivery model known as "telepractice" [4-8]. Telepractice allows speech therapists to deliver their services remotely rather than in person. The World Health Organization (WHO) defines telepractice as the delivery of clinical and rehabilitation services by means of information and communication technology (ICT) with the assistance of telephones, mobile applications, and other videoconferencing platforms in settings in which the patients and their treating physicians are geographically separated from one another over a range of distances, both short and long [3,4,7,9]. In light of the current COVID-19 pandemic, the purpose of this review article is to study the usefulness of using telepractice for speech-language therapy and to discuss potential next steps in the field.

## Review

Methodology

**Figure 1:**
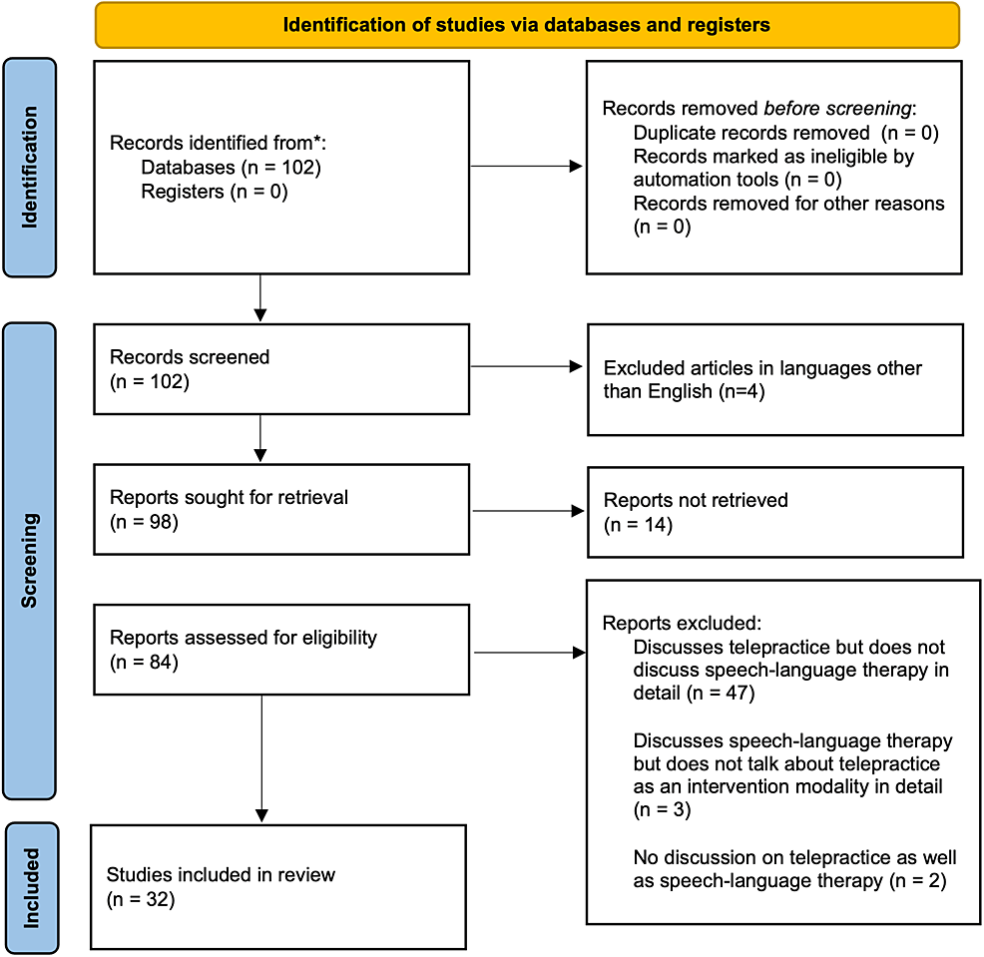
PRISMA flow diagram for the selection of review materials PRISMA: Preferred Reporting Items for Systematic Reviews and Meta-Analyses Credits: Page et al. [10]

A detailed search was done on PubMed and advanced Medical Subject Headings (MeSH) terms, such as "covid," "voice therapy," "speech therapy," "audiologic rehabilitation," "aural rehabilitation," "auditory rehabilitation," "telepractice," and "telemedicine," were used interchangeably and in combination. The inclusion criteria consisted of all articles that were published between the years 2020 and 2022, discussed telepractice and speech-language therapy, were in the English language, and for which PubMed or the publisher provided open access. The articles that were excluded were articles that were in languages other than English, were not retrievable (i.e., not having open access), and discussed either telepractice or speech-language therapy but not both. A total of 102 articles were found, but only 32 of them were chosen to be included because it was determined that they were pertinent. These were selected following the Preferred Reporting Items for Systematic Reviews and Meta-Analyses (PRISMA) guidelines [10]. A comprehensive outline for the selection method is given in Figure 1.

The endpoints of the articles that were included in our review are given in Table 1.

**Table 1:** Details of individual studies that are included

Sr no.	Author	Year	Endpoint	Type of study	Publishing journal
1	Tenforde AS et al. [8]	2020	Patient’s experience of telerehabilitation during COVID-19	Online survey	American Journal of Physical Medicine and Rehabilitation
2	Castillo-Allendes A et al. [9]	2020	Promotion of effective clinical practice for voice assessment and rehabilitation during the COVID-19 pandemic	Literature review	Journal of Voice
3	Dimer NA et al. [11]	2020	Implementation of telepractice in speech-language and hearing therapy for the patients at home	Case report	Scielo-Brazil
4	Tohidast SA et al. [12]	2020	Services provided to treat speech-language disorders in children during COVID-19	Review article	International Journal of Pediatric Otorhinolaryngology
5	Cantarella G et al. [13]	2020	Challenges faced during virtual aural rehabilitation during COVID-19	Review article	Journal of Voice
6	Claridge R et al. [14]	2021	Use of telemedicine for aural rehabilitation	Quantitative (survey) study	International Journal of Telerehabilitation
7	Lam JHY et al. [15]	2021	Perception of parents and students on different modes of delivery of speech-language therapy services	Quantitative (survey) study	Journal of Medical Internet Research
8	Sevitz JS et al. [16]	2021	Challenges faced in telepractice for the management and evaluation of patients with dysarthria	Review article	American Journal of Speech-Language Pathology
9	Terrell EA et al. [17]	2021	Current state of telepractice policy principles and priorities for rehabilitation services	Report	International Journal of Telerehabilitation
10	Hoi KK et al. [18]	2021	Use of telemedicine to complex pediatric otolaryngology during COVID-19 and beyond	Narrative review article	OTOJournal
11	Ransdell LB et al. [19]	2021	Conference on building connections and best practices in telepractice	Report of a conference	International Journal of Telerehabilitation
12	Palomares-Aguilera M et al. [20]	2021	Use of telepractice for providing speech pathology for Spanish-speaking children with cleft palate	Systematic review	International Journal of Pediatric Otorhinolaryngology
13	Al Awaji NN et al. [21]	2021	To explore current situation of speech language and services in Saudi Arabia based on caregivers' perspective	Cross-sectional study	PLOS One
14	Kong AP [22]	2021	To reduce social distress in persons with aphasia residing in remote locations via telerehabilitation	Report	Journal of Speech, Language and Hearing
15	Aazh H et al. [23]	2021	Use of telepractice for tinnitus therapy during COVID-19	Quantitative study	International Journal of Audiology
16	Gefen N et al. [24]	2021	To monitor usage and attitude of online rehabilitation for different therapies and also to decide whether to continue it as full or part time post COVID	Cohort study	International Journal Environmental Research and Public Health
17	Kraljević JK et al. [4]	2020	Preparation for delivery of telepractice by speech-language pathologists (SLPs) in Croatia during COVID-19	Quantitative study	International Journal of Telerehabilitation
18	Pamplona MDC et al. [7]	2020	To study whether SLP intervention by telepractice, speech performance in children with cleft palate	Original article	International Journal of Pediatric Otorhinolaryngology
19	Tomaiuoli D et al. [3]	2021	To evaluate the effectiveness of the Multidimensional, Integrated, Differentiated, Art-Mediated Stuttering Program (MIDA-SP) delivered online for school-age children who stutter	Non-randomized control trial	International Journal of Telerehabilitation
20	Fernandes FDM et al. [25]	2020	Reflections on relevance of using telepractice by SLPs as a part of professional training	Letter to editor	Scielo-Brazil
21	Dorsey ER et al. [26]	2020	5 C's (care, comfort, convenience, cost, and confidentiality) that will build the future of telemedicine	Article commentary	Journal of Parkinson’s Disease
22	Hermes SS et al. [27]	2021	To recognize the barriers faced by SLPs of school in providing telehealth in a rural setting during COVID-19	Cross-sectional study	International Journal of Telerehabilitation
23	Kwok EYL et al. [28]	2022	Experiences of clinicians regarding transition from in-person speech and language therapy services to telepractice	Narrative enquiry	BMC Health Services Research
24	Law J et al. [5]	2021	Teletherapy for children and young individuals with communication difficulties	Systematic review article	International Journal of Language and Communication Disorders
25	Campbell DR et al. [29]	2022	Evolution of telepractice on pediatric SLP services during COVID-19	Quantitative study	American Journal of Speech-Language Pathology
26	Erickson S et al. [30]	2021	Use of telepractice by SLPs for stuttering school-age children	Pilot study	Language Speech and Hearing Services in Schools
27	Cassarino L et al. [31]	2022	Use of telerehabilitation for treatment of post-stroke aphasia during COVID-19	Case report	Journal of Integrative Neuroscience
28	Lee A et al. [32]	2022	Impact of COVID-19 on electropalatography (EPG) research and therapy in Japan	Review article	International Journal of Language and Communication Disorders
29	Southby L et al. [33]	2022	To describe and examine parent views of SLP for children born with cleft palate via teletherapy during COVID-19 in United Kingdom	Quantitative study	Language Speech and Hearing Services in Schools
30	Ziani M et al. [34]	2022	Perception of adolescents and young individuals for providing physical health via telemedicine during COVID-19	Quantitative study	Journal of Patient Experience
31	Lawford BJ et al. [6]	2022	Comparing efficacy and acceptability of allied healthcare delivered via telepractice for adults with disabilities during COVID-19	Cross-sectional national survey	Archives of Physical Medicine and Rehabilitation
32	Bhattarai B et al. [35]	2022	Challenges faced by SLPs delivering telepractice	Cross-sectional survey	Indian Journal of Otolaryngology and Head and Neck Surgery

Discussion

According to the American Speech and Language Association (ASHA), speech-language therapy refers to the therapy that is provided by speech-language pathologists (SLPs) or speech-language therapists (SLTs). The role of an SLP as a healthcare professional is to prevent, assess, diagnose, and treat speech-language disorders, communication disorders, and swallowing disorders and deliver aural rehabilitation to people who are deaf and hard of hearing [3]. Individuals who suffer from severe disorders of expressive or language comprehension, such as autism spectrum disorder, may benefit from SLPs who are trained to provide augmentative and alternative communication systems. The kinds of therapies that are carried out change depending on the age of the individuals being treated; for children, there are language intervention activities and articulation therapies, among others. Adults who develop speech and language disorders as a result of a stroke, injury, or other causes are treated with a variety of approaches, such as the modification of their accents and the performance of exercises for different tongue movements. Therefore, SLPs utilize a wide variety of speech-language therapy procedures and techniques in accordance with the requirements of their patients. These procedures and techniques include aerosol-generating procedures (AGPs), of which the most widely used is "semi-occluded voice therapy exercises" (SOVTEs), which are designed to improve the patient's voice resonance [13]. A wide variety of speech therapy materials are utilized in the treatment of speech and language disorders. For instance, in the case of children, there are a variety of speech-language screeners, bell curve charts, and fun decks, among others. Adults practise things, such as flashcards, tongue twisters, mirror exercises, licking ice cream, and other similar activities.

After the WHO declared that COVID-19 had reached pandemic proportions, governments of different countries took a variety of preventative measures, including a complete lockdown [9,20,22,23,36,37]. Because of this, speech therapy programs, which typically require face-to-face sessions, including touching the patient for some specific procedures or communicating with the help of some unique toys in the case of children, were severely disrupted [6,9,11,13,16,21,25]. This was a problem because speech therapy programs typically require face-to-face sessions. The interruption of speech and language therapies had a negative impact not only on the patients but also on the patient’s families (especially in the case of children), as the parents of these children suffer physically, socially, psychologically, and most importantly economically as a result of the interruption [12]. In the end, this resulted in inadequate parental care [12]. In addition, children who have speech and language pathology should start the necessary therapy as soon as possible because as the child gets older, the neural plasticity decreases, which makes it even more challenging to treat the child. For this reason, the earlier the child starts therapy, the better the outcome [30]. Moreover, persistent stuttered speech has been shown to have a significant impact on the physical, mental, social, and emotional well-being of both adolescents and adults [30]. For these reasons, it has become absolutely essential for the current healthcare system to rapidly transform into a new model for the delivery of healthcare services so that patients can benefit from the change [15,21,33].

Before the pandemic, telepractice was not as commonly used as it is today. However, it is now the only solution for ensuring that continued and uninterrupted healthcare services are provided, which has ultimately led to the rapid adoption and expansion of telepractice [3-8]. The process of providing clinical and rehabilitation services through the use of information and communication technology is referred to as telepractice. The term "telehealth" is frequently used as a synonym for telepractice [3,4,24,35]. Telepractice is utilized not only for the purpose of diagnosing and treating a variety of conditions, but it also has the potential to be used as a platform for the delivery of continued education to healthcare professionals working in a variety of settings [4]. The synchronous, asynchronous, and hybrid types of telepractice are shown in Table 2.

**Table 2:** Types of telepractice

Type of telepractice	Description	
Synchronous	These are interactive services that occur in real time between the patient and clinician [4,8,19,28].	
Asynchronous	It includes the storing of materials that the patient or clinician can access at a later time, such as voice recordings and video recordings [4,8,9,19,28].	
Hybrid	It is the combination of both synchronous and asynchronous methods [4,8,9,19,28].	

During the pandemic, many medical professionals started turning to telepractice services as a means of overcoming, in some way, the disruption that the pandemic caused in the delivery of healthcare services. Telepractice, which is easy to access and was given the name "attend anywhere web-based service" due to the fact that it allows patients to receive treatments regardless of their location, has thankfully proven to be the only method by which SLPs have been able to continue their sessions in order to limit the spread of infection and continue to provide ongoing care [8,23]. Other benefits included limiting the geographical barriers, reducing the cost of travel, saving time, and thus serving a great purpose in situations where time was a significant issue [3]. It has been found that conducting speech-language therapy sessions via telepractice is on par with running those sessions in person when it comes to the prevention, assessment, diagnosis, and management of various disorders [3]. Patients who had participated in telepractice sessions reported feeling more satisfied with their care overall, and they noted that the sessions had a sedating and relaxing impact on their minds. Patients were given the same sense of comfort that they would receive from in-person visits, and they anticipated receiving more of these types of consultations in the future, even after the pandemic has been contained [8,11,19,24].

The telepractice sessions for the provision of speech-language therapy has shown to have greater overall satisfaction, which was rated using a five-point Likert scale in a study done in 2020 [8]. The response options ranged from excellent to poor, which included the options excellent, very good, good, fair, and poor, with the majority of the responses falling under the excellent and very good categories [8]. The satisfaction is particularly more among women as compared to men; studies suggest that since women have to take care of their children and have to perform other tasks, such as managing the house, so they found these telepractice services to be more beneficial as compared to the traditional healthcare service delivery model as they can easily show their child to an SLP without even going out of their home [8]. It has been seen that two-thirds of women use this web-based health model on weekends and holidays when the hospitals are said to be closed, as stated in one of the reports [8]. Meanwhile, telepractice sessions are being accepted by more number of people some of the important steps while setting up the session includes checking the Internet connectivity before starting the session, selecting a room that is quiet and has adequate lighting, using an external microphone for proper communication, speaking clearly during the session, using simple sentences that are brief and easy to understand by the patient, maintaining an appropriate posture in front of the camera so that the face is clearly visible, using subtitles so that the patient can understand when the Internet is slow [9].

In addition, there are many variations of the Health Insurance Portability and Accountability Act (HIPAA) or the Family Educational and Privacy Act (FERPA) - video conferencing programs that are easy to use and have been made available. Moreover, there have been many improvements in the provision of high-speed broadband connections, which have allowed everyone to use telepractice sessions efficiently and effectively [29]. As a result, the benefits of telepractice can be summed up as: ease of access to medical care, increased levels of comfort and convenience, increased levels of confidentiality for patients and their families, and, last but not the least, a decreased likelihood of spreading infections [26].

Although there are many benefits associated with telepractice, there are also some drawbacks that cannot be ignored. These drawbacks include lack of equipments, lack of proper skills among the SLPs, lack of adequate access to the Internet in some areas, and lack of access to hands-on skills (which means that it does not have the humaneness that builds a bond between the patient and clinician), and other barriers include reimbursement policies [4,18,24,26,27,31,33,34,37].

It has also been discovered that a lack of familiarity with digital technology is one of the primary reasons for the decline in the utilization of telepractice [4]. It was found that the overall efficacy of providing telepractice decreases as a person's age increases. In addition, it was discovered that people over the age of 65 have a digital literacy rate that is 10 percentage points lower than that of younger people [4]. Certain patients, particularly in Croatia, found that face-to-face therapy sessions were more effective than telepractice in achieving their treatment goals [4]. There have been a few steps taken to reduce these limitations; for instance, in one of the studies, distributions of lesson kits, namely, "MED-EL KITS," were done. These kits can be easily downloaded by the practitioner in a PDF format directly from MED-EL Professionals Blog. The major problems reported by clients receiving telepractice were a lack of proper educational skills, reimbursement, and no proper access to the Internet [14]. After going through these kits, the SLPs felt more confident while providing the telepractice session, and also, the patient's level of satisfaction increased to a significantly higher level [14]. With regard to reimbursement, numerous organizations, such as the American Occupational Therapy Association (AOTA), American Physical Therapy Association (APTA), ASHA, and American Telemedicine Association (ATA) are working tirelessly to urge the government to pay an equivalent sum to all telepractice service providers [17]. 

To ensure the sustainability of speech-language therapy via telepractice, several organizations, namely, the AOTA, APTA, ASHA, and ATA, have joined hands with one another during the Telemedicine Awareness Week (TAW) [17] to ensure that telepractice services sustain even after the COVID-19 pandemic. They focused on certain specific priorities, such as not placing any limitations on the provision of telepractice services based on the type of provider and also encouraging the independence of providers, supporting the bipartisan Telepractice Access Act, which would allow us to expand the type of practitioners that would be eligible to provide a telerehabilitation service and also allowing the providers to become permanently authorized persons to provide telepractice services under the Medicare program [17]. Some other priorities included insisting the government not to impose any location restrictions on patients, allowing them to have convenient access to the service wherever they are, including at home, ensuring that all providers of telepractice services receive the same amount of money for their services [17].

It is imperative that these steps should be taken in order to avoid the "telepractice cliff" for speech therapy services [17]. If these steps are not taken within the appropriate time, then telepractice would surely come to an end, which is something that should be avoided at all costs as telepractice has become a lifeline for a great number of people [17].

## Conclusions

It was during this novel coronavirus pandemic period that a great number of studies were carried out to find not only the usefulness, advantages, and disadvantages of telepractice but also the possibilities of telepractice being accepted as a future model for the delivery of healthcare. Overall, the studies show that patients are just as satisfied with telepractice as they were with in-person visits. This finding is supported by the observations made by the researchers. In spite of this, there is a pressing need for additional studies to be conducted on the environmental and ethical concerns raised by the utilization of telepractice in speech-language therapy.
